# Receptors expressions on peripheral lymphocytes and CD4^+^CD183^+^ as a diagnostics biomarker for rheumatoid arthritis: A case–control study in Ghana

**DOI:** 10.1002/iid3.976

**Published:** 2023-08-29

**Authors:** Samuel Asamoah Sakyi, Tonnies Abeku Buckman, Kwame Yeboah‐Mensah, Ebenezer Senu, Alfred Effah, Daniel Antwi‐Berko, Dzifa Dey, Maxwell H. Antwi, Joseph Yorke, Andy O. Boateng, Akwasi M. Addei, Muniru M. Tanko, Richard Boateng

**Affiliations:** ^1^ Department of Molecular Medicine, School of Medicine and Dentistry Kwame Nkrumah University of Science and Technology Kumasi Ashanti Region Ghana; ^2^ Department of Medical Laboratory Science University of Energy and Natural Resources Sunyani Ghana; ^3^ Department of Medical Laboratory Sciences KAAF University College Accra Ghana; ^4^ Department of Medicine, School of Medicine and Dentistry, Komfo Anokye Teaching Hospital Kwame Nkrumah University of Science and Technology Kumasi Ghana; ^5^ Neurochemistry Laboratory, Department of Clinical Chemistry VU University Medical Center (VUmc) Amsterdam The Netherlands; ^6^ Department of Medicine and Therapeutics, Korle‐Bu Teaching Hospital University of Ghana Medical School Accra Ghana; ^7^ Department of Medical Laboratory Sciences Koforidua Technical University Koforidua Ghana; ^8^ Department of Surgery, School of Medicine and Dentistry Kwame Nkrumah University of Science and Technology Kumasi Ghana; ^9^ Directorate of Surgery Komfo Anokye Teaching Hospital Kumasi Ghana; ^10^ Department of Biological Sciences Kwame Nkrumah University of Science and Technology Kumasi Ashanti Region Ghana; ^11^ Department of Immunology and Immunodiagnostics University for Development Studies Tamale Northern Region Ghana; ^12^ Department of Clinical Microbiology Komfo Anokye Teaching Hospital Kumasi Ashanti Region Ghana

**Keywords:** CD4^+^CD183^+^, chemokine receptors, diagnostics biomarker, disease activity score, receptor expression, rheumatoid arthritis

## Abstract

**Background:**

T cell receptors play important roles in the development and progression of rheumatoid arthritis (RA). Their involvement has been reported in inflammatory autoimmune diseases. However, their role in predicting RA is still under exploration. This study evaluated the expression of CD183 (CXCR3) receptors on T‐cells and other relevant biomarkers for detecting RA and determine their relationship with disease activity.

**Methods:**

This unmatched case–control study included 48 newly diagnosed RA patients and 30 apparent healthy controls from the orthopedic units of Komfo Anokye Teaching Hospital (KATH), Kumasi and Korle‐Bu Teaching Hospital (KBTH), Accra, Ghana. Sociodemographic data was obtained, and blood samples were also collected and processed for flow cytometric analysis. Statistical analyses were done using SPSS version 26.0 and R programming language. *p* < .05 was considered statistically significant.

**Results:**

This study found a significant difference in age group (*p* < .0001), marital status (*p* = .0210), occupation (*p* = .0140), educational level (*p* = .0210) and religion (*p* = .0100) between RA patients and healthy controls. Moreover, hemoglobin level (*p* = .0010), waist circumference (*p* < .0001) and hip circumference (*p* = .0040) were significantly different between RA patients and controls. RA patients had significantly lower levels of CD4^+^CD183^+^ compared with the control group (*p* < .001), and was positively correlated with DAS score (*r* = .0397, *p* = .789). In Receiver Operator Characteristics analysis, CD4^+^CD183^+^ could significantly detect RA with a high area under the curve (AUC = 0.687, *p* = .018). At a cut‐off of 0.082, CD4^+^CD183^+^ was the best receptor biomarker for detecting RA with a sensitivity of 90.0%, specificity of 25.9%, a positive predictive value of 69.2%, and a negative predictive value of 58.3%.

**Conclusion:**

CD4^+^CD183^+^ best predict RA and is positively correlated with disease activity. CD4^+^CD183^+^ could serve as diagnostics and disease‐monitoring biomarker for RA; however, it demonstrates low specificity. Future studies should be directed on CD4^+^CD183^+^ and other biomarkers to augment their diagnostics performances and routine management in RA.

## 1 INTRODUCTION

Rheumatoid arthritis (RA) is a chronic systemic autoimmune disease characterized by persistent development of pathologic self‐attacking immune response affecting the musculoskeletal system resulting in joints deformation and severe pain. Though the exact pathogenesis of RA has not been thoroughly explicated,^1^ cluster of differentiation positive 4 (CD4^+^) T cells are thought to play a crucial role, as it stimulates proliferation and differentiation of B‐lymphocytes^2^ and participate in the induction and propagation of inflammatory responses by secreting pro‐inflammatory cytokines, growth factors, and interferons.^3^, ^4^ Moreover, previous study has reported severity of arthritis was associated with reduce Foxp3 expression,^5^ and low CD294^+^ expression in RA.^6^ Moreover, CD127^+^ has been linked to human inflammatory monocytes with direct relevance for inflammatory diseases.^7^

Movement of CD4^+^ T cells, and co‐existent their inflammatory mediators into the synovial fluid contribute to initiation, propagation, and maintenance of chronic inflammation of synoviocytes.^8^ The migration of CD4^+^ T cells as well as other immune cells to lymphoid or rheumatoid synovium is dependent on several factors including their chemokine receptors expression profile.^9^ There is now evolving evidence that the complex chemokine network is poorly regulated in these diseases, although many more studies will be necessary to get a thorough understanding of the underlying mechanisms.^10^ Among the large chemokine and chemokine receptor families, CXCR3 and CXCR3‐binding chemokines are likely to be key players in the maintenance and amplification of the autoimmunity‐related inflammatory processes.^10^, ^11^ Coincidently, CXCR3 has become an attractive target for the drug discovery community, and several families of molecules with potential clinical applications, such as specific blocking antibodies, protein‐based antagonists, and small chemical compounds have been developed.^12^, ^13^ Systematic review of studies that have been done mainly on human samples like synovial fluid, synovial tissue and blood collected from RA patients have provided an overall idea of the implication of various chemokine receptors including CXCR3 in this chronic inflammatory disorder, which mainly affects joints.^13^, ^14^ The inflamed synovial tissue of RA patients is characterized by a massive infiltration of immune cells, among which Th1‐type T CD4+ cells are predominant.^15^

One of the chemokine receptors that are highly expressed on the surface of Th1‐polarized T cells has been reported to be CXCR3.^15^ CXCR3 is a seven‐transmembrane G protein‐coupled receptor for CXCL9, CXCL10, and CXCL11.^16^, ^17^ CXCR3 is expressed by endothelial cells, mast cells, T cells, and fibroblast‐like synoviocytes (FLS).^18^ The increased manifestation of CXCR3 and CCR5 chemokine ligands have been reported in a study by Turner et al.^19^ to be followed by the recruitment of CXCR3‐ and CCR5‐positive T cells which is suggestive of an important key role for these chemokine receptors in T cell‐mediated tissue damage as seen in human inflammatory diseases, including RA.

It has again been evident that, approximately 90% of CD4+ T cells present in the synovial fluid of RA patients when intensely stained for immunofluorescence express CXCR3 receptor. Aside the T cells, CXCR3‐expressing mast and plasma cells are also seen in the infiltrated synovial tissue of RA patients.^10^ This is a strong indication that both cell types may add to the sustenance of the inflamed state in arthritic lesions. When compared with osteoarthritis, studies have indicated a significant higher level of CXCR3‐binding chemokines in RA patients, both in synovial fluid and tissue.^11^, ^13^

CXCR3 pathway plays a key role in joint inflammation occurring in RA as it has been demonstrated that lipoxygenase, cyclooxygenase and TNF‐α inhibitors which serve as analeptic molecules inhibit the synthesis of CXCR3 ligands by synovial fibroblasts and alter the migration of CXCR3+ T cells.^20^, ^21^ Some studies have identified synovial fibroblasts as the main producers of the three CXCR3‐agonistic chemokines CXCL9, 10, and 11 in response to the synergistic effect of interferon‐γ (IFN‐γ) and tumor necrosis factor (TNF‐α).^21^ Previous studies on CXCR3 have been shown to increase disease activity in RA. Furthermore, one study demonstrated that inhibition of particular chemokines reduces inflammation and subsequent damage to cells.^22^ Investigating chemokines and other receptor expressions for their role in exacerbating inflammation and damage to synoviocytes could be useful as potential biomarker for diagnoses and monitoring RA patients. However, their role in RA and predicting diseases activity is still under exploration. The aim of this study was to investigate the expression pattern of CD183 (CXCR3) chemokine receptors on peripheral lymphocytes and other relevant biomarkers in RA patients including a prostaglandin D2 receptor (CD4+ CD294+), an interleukin‐7 receptor‐α (CD4+ CD127+), and a transcription factor (CD4+ FOXP3+) as diagnostics biomarkers for detecting RA, and determine their relationship with disease activity. This would contribute to the understanding of the immunological mechanisms underlying RA and potentially identify novel biomarkers for RA disease detection and monitoring.

## 2 MATERIALS AND METHODS

### 2.1 Study design and study setting

This unmatched case–control study recruited RA patients from the orthopedic units of Komfo Anokye Teaching Hospital (KATH), Kumasi and Korle‐Bu Teaching Hospital (KBTH), Accra, Ghana. KATH, the second largest hospital in Ghana, is a 1200‐bed facility in the Kumasi Metropolis. KBTH is the largest and third largest health facility in Ghana and Africa, respectively, with over 2000‐bed capacity. The orthopedic units of both hospitals provide health care services to both in‐ and out‐patients with rheumatologic and autoimmune conditions. The unmatched case–control study adopted in the present study was due to rarely diagnosed RA cases and available controls to be recruited for the study.

### 2.2 Sample size calculation

The sample size was obtained using the online sample size calculator for unmatched case–control study (https://www.openepi.com/SampleSize/SSCC.htm). In this unmatched case–control study, 48 RA patients (cases) and 30 healthy controls were recruited.

### 2.3 Participants recruitment

#### 2.3.1 Inclusive and exclusion criteria

**Figure 1:**
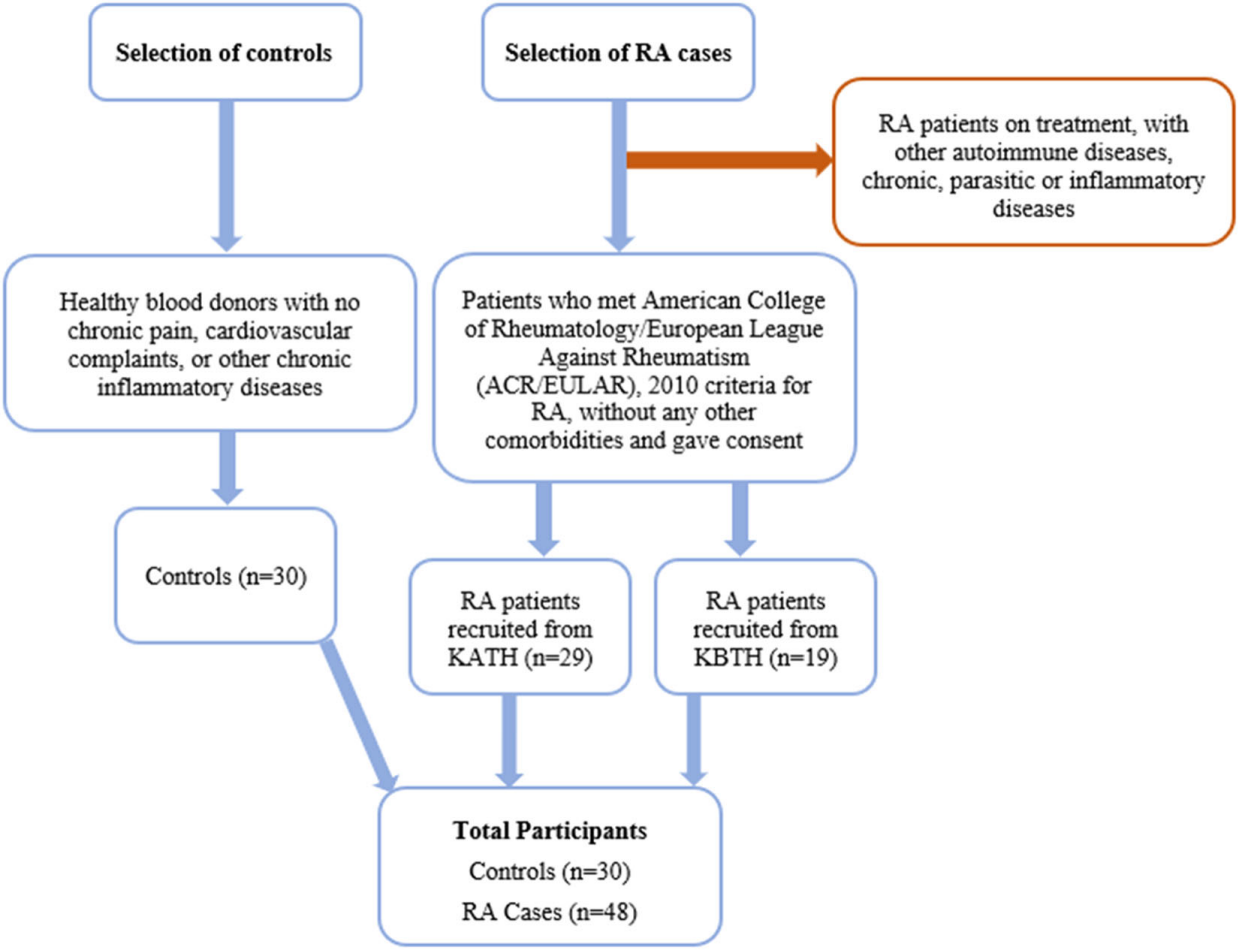
Schematic flowchart for recruitment of participants into the study. RA, rheumatoid arthritis.

A total of 48 consecutive consenting newly diagnosed RA patients, 29 from KATH and 19 from KBTH, were included as cases in this study. Diagnosis of RA at both clinics were according to the American College of Rheumatology/European League Against Rheumatism (ACR/EULAR), 2010 criteria for RA.^23^ RA classification criteria were done by two independent Rheumatologists. All patients were prednisolone‐naïve. However, RA patients who did not meet the ACR/EULAR 2010 criteria were excluded from the study. Patients with other autoimmune diseases, those who received treatment with DMARDs, glucocorticoids, or/and vitamins, women during pregnancy, and persons with diabetes mellitus or metabolic syndrome were also excluded from the study (Figure 1).

#### 2.3.2 Selection of controls

Healthy blood donors with no chronic pain, cardiovascular complaints, chronic inflammatory diseases, malaria, TB, or parasitic infection and who gave informed consent were recruited and used as control (Figure 1).

### 2.4 Ethics approval and consent to participate

Ethical approval for this study was obtained from the Committee on Human Research, Publication and Ethics (CHRPE) of the School of Medical Sciences, Kwame Nkrumah University of Science and Technology (CHRPE/AP/003/16) and the institutional review board of KATH and KBTH. Written informed consent was obtained from all participants who opted to participate after the aims and objectives of the study had been explained to them. Participation was voluntary, and respondents were assured that the information obtained was strictly for research and academic purposes only and were guaranteed the liberty to opt out from the study at their own convenience.

### 2.5 Questionnaire administration, blood pressure, and anthropometric evaluation

Questionnaires were administered to obtain sociodemographic data from the participants. Data collected include age, sex, marital, educational, and employment status. Additional clinical data relevant to the study were retrieved from the hospital's archives. Weight was measured in the upright position to the nearest 0.1 kg using a calibrated balance beam scale. Height was measured (subjects stood erect, barefoot, with feet together, looking forward) to the nearest 0.1 m using a measuring tape. Body mass index (BMI) was calculated using the equation = weight (kg)/height (m)^2^. Blood pressure was measured with an automated blood pressure apparatus (Omron MX3‐Omron Matsusaka Co., Ltd.) from the right arm after the subject had been made to sit for at least 5 min. The average of the two readings taken 5 min apart was recorded.

### 2.6 Blood sample processing, cell preparation, and flow cytometric analysis

Eight milliliters (8 mL) of venous blood was drawn from each RA patients and healthy controls. Four milliliters (4 mL) was dispensed into Ethylenediamine tetra‐acetic acid (EDTA) tubes for the estimation of erythrocyte sedimentation rate (ESR) using the Westergren method. The remaining 4 mL was dispensed into tubes containing heparin for the isolation of peripheral mononuclear cells (PBMCs) using the Ficoll‐Paque density gradient centrifugation (Biochrom). Briefly, the whole blood was poured into the Ficoll containing tubes and centrifuged at 1500 rpm for 30 min at 4°C with no brakes. Cells were suspended in Roswell Park Memorial Institute (RPM) 1640 medium, supplemented with 0.5% Dimethyl Sulfoxide (DMSO) and 0.5 mL Fatal Bovine Serum (Biochrom) at a density of 0:5 × 104 cells/mL. Collected PBMCs were stored at −80°C until further analysis.

Before flow cytometric assessment of intracellular cytokine expression, frozen PBMCs were thawed in 37°C water bath for 15 min. The cells were washed in phosphate‐buffered saline (PBS) and suspended in a small amount of PBS. Hundred microliters (100 μL) of the resultant cell suspension was pipetted into 96‐well plates, followed by centrifugation of plates at 1500 rpm for 5 min. About 180 μL supernatant was pipetted and discarded after which cells were incubated for 10 min at room temperature in the dark. Cells were fixed and permeabilized using fixation/permeabilization reagent from BioLegend^24^ followed by intracellular staining for CD4+ T cell expression of APC anti‐CD183/CXCR3 (catalog 353708; Biolegend), Alexa647anti‐FOXP3 (catalog 320214p; Biolegend), CD294‐PE (catalog 350106; Biolegend), and CD 127‐ PE‐Cy7 (catalog 300924; Biolegend) human antibodies. Cells were gated on lymphocyte population and CD4+ T cells, and analysis of percentage of cells expressing biomarkers was done on the gated population using the Accuri C6 Flow Cytometer (Accuri Cytometers Inc.). According to information supplied by the manufacturer, the BioLegend reagents have been validated to be of higher sensitivity and specificity accordingly to the manufacturer's protocol thereby reducing inconsistent results to be expected in this study.^24^

### 2.7 Assessment of disease activity

Disease activity was assessed based on the disease activity score (DAS) in 28 joints. Estimation was based on clinical parameters (tender and swollen joint counts), visual assessment scale, and laboratory biomarkers of inflammation (ESR).

RA patients were grouped based on DAS28 scores; low disease activity (DAS28 ≤ 3:2), moderate disease activity (3:2 < DAS28 ≤ 5:1), and high disease activity (DAS28 > 5:1).^25^

### 2.8 Data management and statistical analyses

Data obtained were entered, cleaned and coded in Microsoft Excel 2019 software. R Language for Statistical computing^26^ and Statistical Package for Social Sciences (SPSS release 26.0, Copyright ©SPSS Inc.) were used for statistical analysis of the data. Parametric continuous variables were expressed as mean (± standard deviation) whilst categorical variables were presented as frequency and percentages. Chi‐square test statistics was used to dertermine sociodemographic factors associated with RA. Comparison between RA cases and controls were done using independent sample t‐test for parametric continuous variables and Mann–Whitney *U* test for non‐parametric continuous variables, with *p* < .05 considered as statistically significant. Correlation analysis was performed between study variables and receptor expressions in RA patients. The receiver operating characteristic (ROC) curve was used in evaluating the diagnostic performance of CD4+CD294+, CD4+CD127+, CD4+FOXP3+, and CD4^+^CD183^+^ for detection RA.

## 3 RESULTS

### 3.1 Baseline characteristics of study participants

A total of 78 participants, consisting of 48 RA cases and 30 healthy controls were included in the analysis. Majority of the cases were within the age group of 50 and above compared with the controls. There was statistically significant difference between the ages of the cases and the controls (*p* < .0001). Considering marital status, most of the cases were married (62.5%), whilst few of the controls were married (56.7%), this was statistically significant (*p* = .0210). Also, there was a significant difference in occupation between the two study groups, since majority of the cases had informal occupation (64.6%) compared with the controls (53.3%) (*p* = .0140). Moreover, about have of the RA cases had secondary education (52.1%) whilst few of the controls had secondary education (20.0%). The study found statistically significant difference in educational level between the cases and the controls (*p* = .0210). For religious background, majority of the cases were Christians (95.8%) compared with controls (26.7%) (*p* = .0100). Furthermore, most of the RA cases were anemic compared with the healthy controls (*p* = .0010). The study found significant difference in the mean waist circumference (cm) of the cases compared with the controls [35.77 (30.47−41.07) vs. 31.57 (27.99−35.15) cm, *p* < .0001]. Similarly, there was a significant difference in mean hip circumference of cases and controls [40.15 (34.20–46.10) vs. 36.93 (33.39–40.47) cm, *p* = .0040].

However, there was no significant difference in male and female proportion between the two study groups (*p* = .0510). Although majority of the cases were obese (47.9%) compared with the cases, no significant difference was observed for BMI status between cases and the controls (*p* = .0950). Similarly, there was no significant difference in ethnicity between the two study groups (*p* = .6910). Moreover, the mean difference of systolic or diastolic blood pressure of cases was similar to that of the controls, with *p* values of .8460 and .8930, respectively (Table 1).

**Table 1:** Sociodemographic characteristics of rheumatoid arthritis patients and healthy controls.

Variable	Controls (*n* = 30)	Cases (*n* = 48)	*p* Value
Age group (years)			**<.0001**
20–29	7 (23.3)	2 (4.2)	
30–39	12 (40.0)	10 (20.8)	
40–49	11 (36.7)	8 (16.7)	
50–59	0 (0.0)	15 (31.3)	
60–75	0 (0.0)	13 (27.1)	
Gender			.0510
Male	11 (36.7)	8 (17.0)	
Female	19 (63.3)	39 (83.0)	
Marital Status			**.0210**
Single	13 (43.3)	9 (18.8)	
Married	17 (56.7)	30 (62.5)	
Divorced	0 (0.0)	1 (2.1)	
Widowed	0 (0.0)	8 (16.7)	
Ethnicity			.6910
Akan	22 (73.3)	27 (57.4)	
Ewe	2 (6.7)	7 (14.9)	
Ga	3 (10.0)	7 (14.9)	
Krobo	1 (3.3)	2 (4.3)	
Northerner	2 (6.7)	4 (8.5)	
Occupation			**.0140**
Unemployed	1 (3.3)	5 (10.4)	
Formal	13 (43.3)	7 (14.6)	
Informal	16 (53.3)	31 (64.6)	
Retired	0 (0.0)	5 (10.4)	
Educational level			**.0210**
No formal education	3 (10.0)	2 (4.2)	
Primary	9 (30.0)	13 (27.1)	
Secondary	6 (20.0)	25 (52.1)	
Tertiary	12 (40.0)	8 (16.7)	
Religion			**.0100**
Christian	23 (26.7)	46 (95.8)	
Muslim	7 (23.3)	2 (4.2)	
BMI			.0950
Normal	8 (26.7)	13 (27.1)	
Overweight	14 (46.7)	12 (25.0)	
Obese	8 (26.7)	23 (47.9)	
Haemoglobin			**.0010**
Normal	24 (80.0)	20 (41.7)	
Anemic	6 (20.0)	28 (58.3)	
Waist Circumference (cm)*	31.57 ± 3.58	35.77 ± 5.30	**<.0001**
Hip Circumference (cm)*	36.93 ± 3.54	40.15 ± 5.95	**.0040**
Systolic Blood Pressure (mmHg)*	128.90 ± 6.88	129.35 ± 13.63	.8460
Diastolic Blood Pressure (mmHg)*	82.83 ± 5.17	82.60 ± 9.74	.8930
ESR	–	45.92 ± 25.98	–
DAS Score	–	3.17 ± 1.07	–

*Note*: *p* Value < .05 and bolded means statistically significant. *p*‐values were computed by Chi‐square test; however, *means data presented as mean and standard deviation and *p*‐value computed by independent sample t‐test.

Abbreviations: BMI, body mass index; DAS, disease activity score; ESR, erythrocyte sedimentation rate.

### 3.2 Comparison between receptors expressions among RA patients and healthy controls

**Figure 2:**
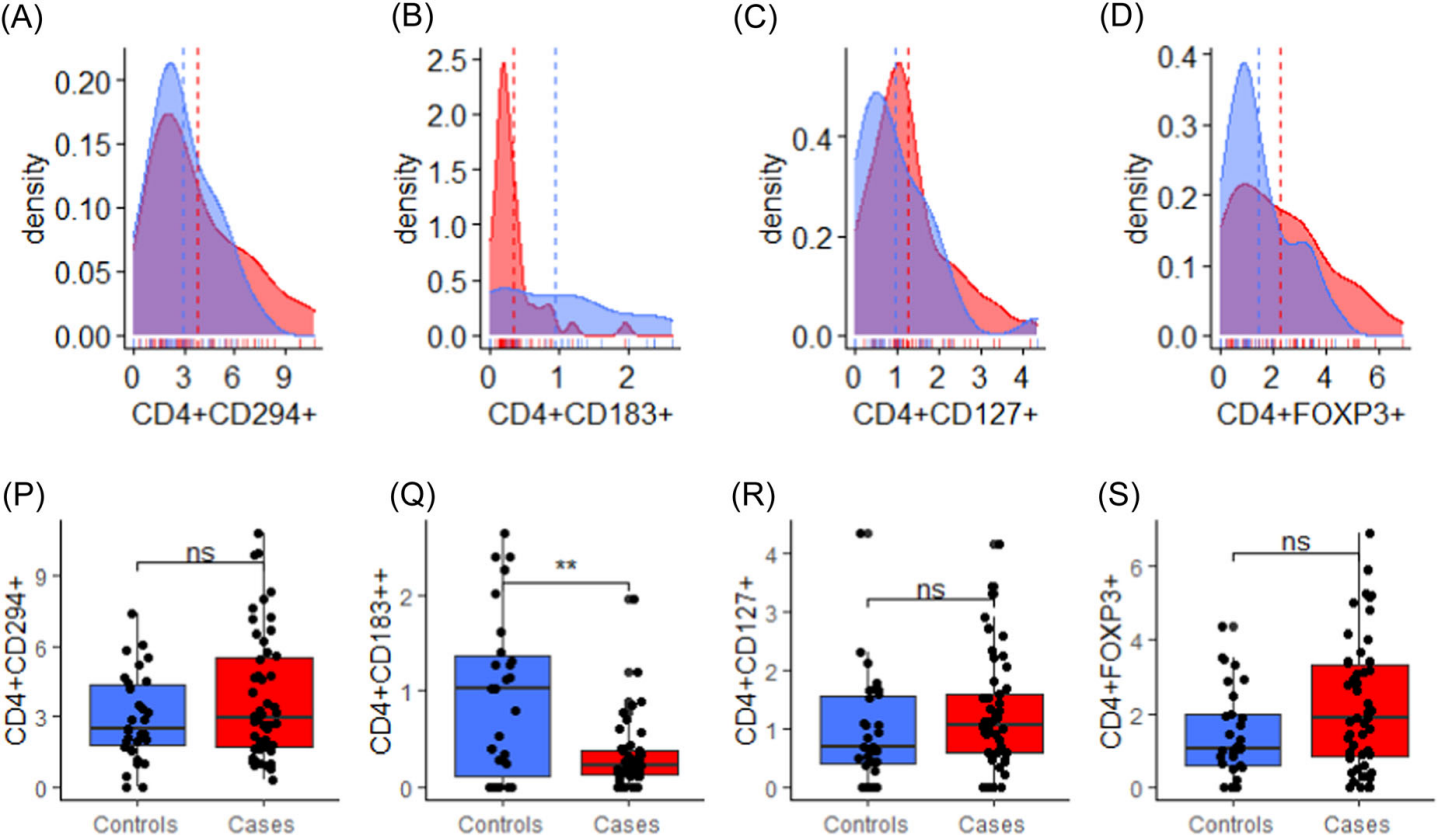
Comparison between receptors expressions among rheumatoid arthritis patients and healthy controls; ns = *p* > .05, *p < .05, **p < .01, ***p < .001; *p*‐values computed by Mann‐Whitney U‐test; (A–D) represent density plot (A‐CD4+CD294+, B‐CD4+CD183+, C‐CD4+CD127+, D‐CD4+FOXP3+); (P–S) represent box and whisker plot (P‐CD4+CD294+, Q‐CD4+CD183+, R‐CD4+CD127+, S‐CD4+FOXP3+).

Figure 2 displays the receptors expressions profile of the study groups. RA patients had significantly lower levels of CD4^+^CD183^+^ compared with the control group (*p* < .001) (Figure 2Q). However, there was no statistically significant difference in the levels of CD4+CD294+, CD4+CD127+ or CD4+FOXP3+ between the two study groups, although the levels of these chemokines were slightly higher in RA patients compared with healthy controls (*p* > .05) (Figure 2).

### 3.3 Correlation between receptors expressions and detecting DAS among RA patients

**Figure 3:**
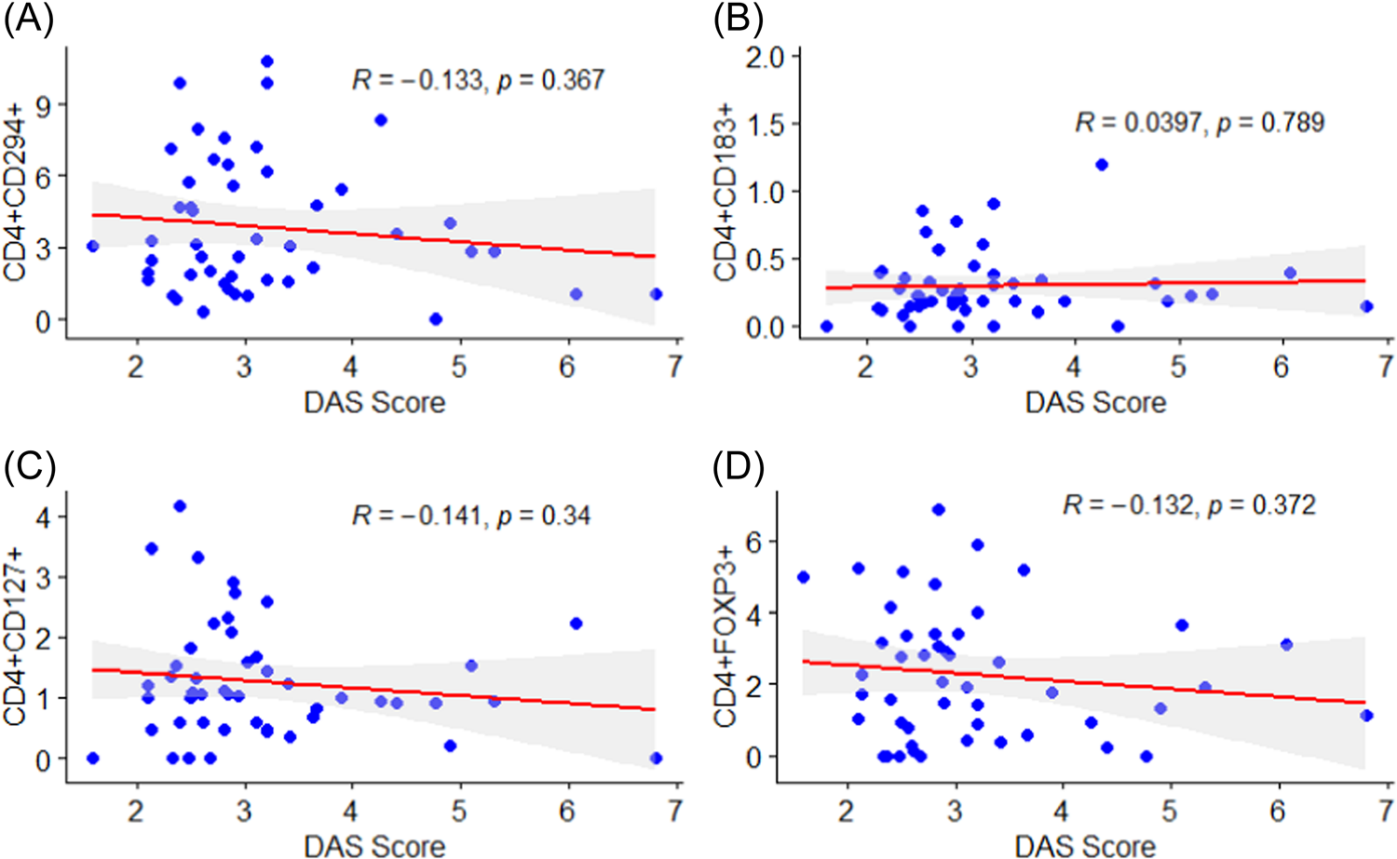
Correlation between receptors expressions and disease activity score (DAS) among rheumatoid arthritis patients, (A) CD4+CD294+, (B) CD4+CD183+, (C)‐CD4+CD127+, (D) CD4+FOXP3+.

Depicted in Figure 3, are the Pearson correlations between receptors expressions and DAS among RA patients. There were insignificant negative correlations between CD4^+^CD294^+^ (*r* = −0.133, *p* = .367), CD4^+^CD127^+^ (*r* = −0.141, *p* = .340), CD4^+^FOXP3^+^ (*r* = − 0.132, *p* = .372) and DAS score (Figure 3A,C,D).

On the contrary, the study found positive correlation between CD4^+^CD183^+^ and DAS score. However, this was statistically insignificant (*r* = .0397, *p* = .789) (Figure 3B).

### 3.4 Diagnostics performance of the various receptor expressions in RA

**Figure 4:**
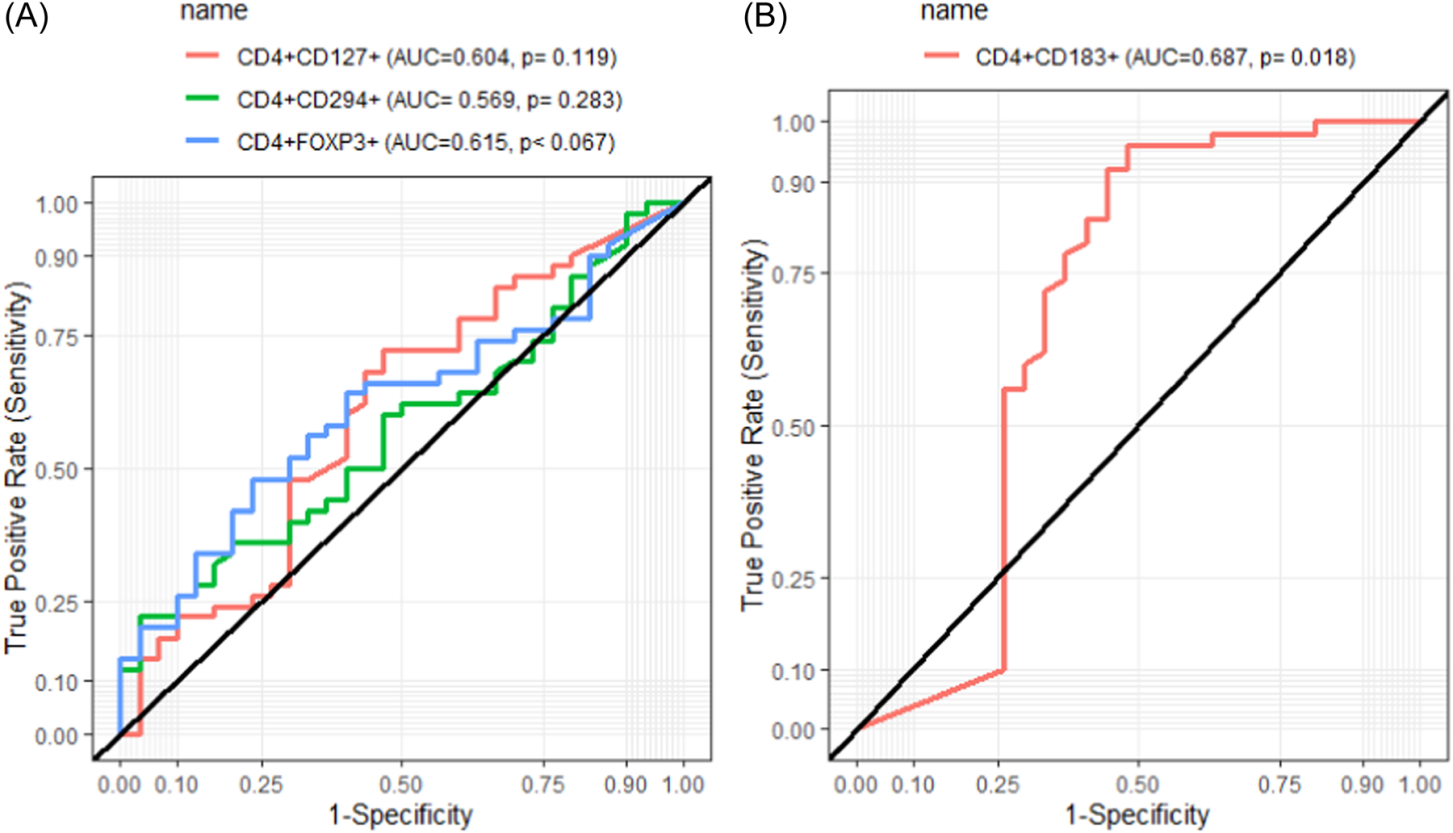
The receiver operating characteristics (ROC) curves of CD4+CD294+, CD4+CD127+, CD4+ FOXP3+, and CD4^+^CD183^+^ for detecting rheumatoid arthritis, (A) CD4+CD127, CD4+CD294+, and CD4+FOXP3+; (B) CD4+CD183+.

The Receiver Operator Characteristics analysis was used to evaluate the diagnostic performance of receptors expressions on peripheral lymphocytes in predicting RA. CD4^+^CD183^+^ could significantly predict the disease activity in RA patients with a very high area under the curve (AUC = 0.687, *p* = .018) (Figure 4B). However, CD4+CD294+ (AUC = 0.569, *p* = .283), CD4+CD127+ (AUC = 0.604, *p* = .119) and CD4+FOXP3+ (AUC = 0.615, *p* = .067) could not significantly predict RA (Figure 4A).

At a cut‐off of 0.082, CD4^+^CD183^+^ was the best receptor for detecting RA with a sensitivity of 90.0%, specificity of 25.9%, a positive predictive value of 69.2%, and a negative predictive value of 58.3%. At a cutoff of 4.170, the CD4+CD127+ was 100.0% sensitive but less specific (3.30%). At a cut‐off of 1.860, CD4+CD294 was 73.3% specific but less sensitive (30.0%). Moreover, at a cut‐off of 0.490, CD4+FOXP3+ was 83.3% specific but less sensitive (22.0%) (Table 2).

**Table 2:** Diagnostics performance of receptor expressions biomarkers for detection rheumatoid arthritis.

Receptor expressions	Cut‐off	Sensitivity (95% CI)	Specificity (95% CI)	PPV	NPV	LR +	LR−
CD4+CD294+	1.860	30.0 (19.1–43.9)	73.3 (55.3–85.9)	65.2	38.6	1.13	0.96
CD4^+^CD183^+^	0.082	90.0 (78.1–96.0)	25.9 (13.1–45.0)	69.2	58.3	1.22	0.39
CD4+CD127+	4.170	100 (91.3–100.0)	3.30 (0.00–18.4)	63.3	100.0	1.03	0.00
CD4+FOXP3+	0.490	22.0 (12.7–35.5)	83.3 (65.8–93.0)	68.8	39.1	1.32	0.94

*Note*: At a cut‐off of 0.082, CD4^+^CD183^+^ was the best receptor biomarker for detecting rheumatoid arthritis with a sensitivity of 90.0%, specificity of 25.9%, a positive predictive value (PPV) of 69.2%, and a negative predictive value of 58.3%.

Abbreviations: CD4, cluster of differentiation 4; CI, confidence interval; LR−, negative likelihood ratio, LR+, positive likelihood ratio; NPV, negative predictive value; PPV, positive predictive value.

### 3.5 Correlation between waist circumference, hip circumference and receptors among RA patients

**Figure 5:**
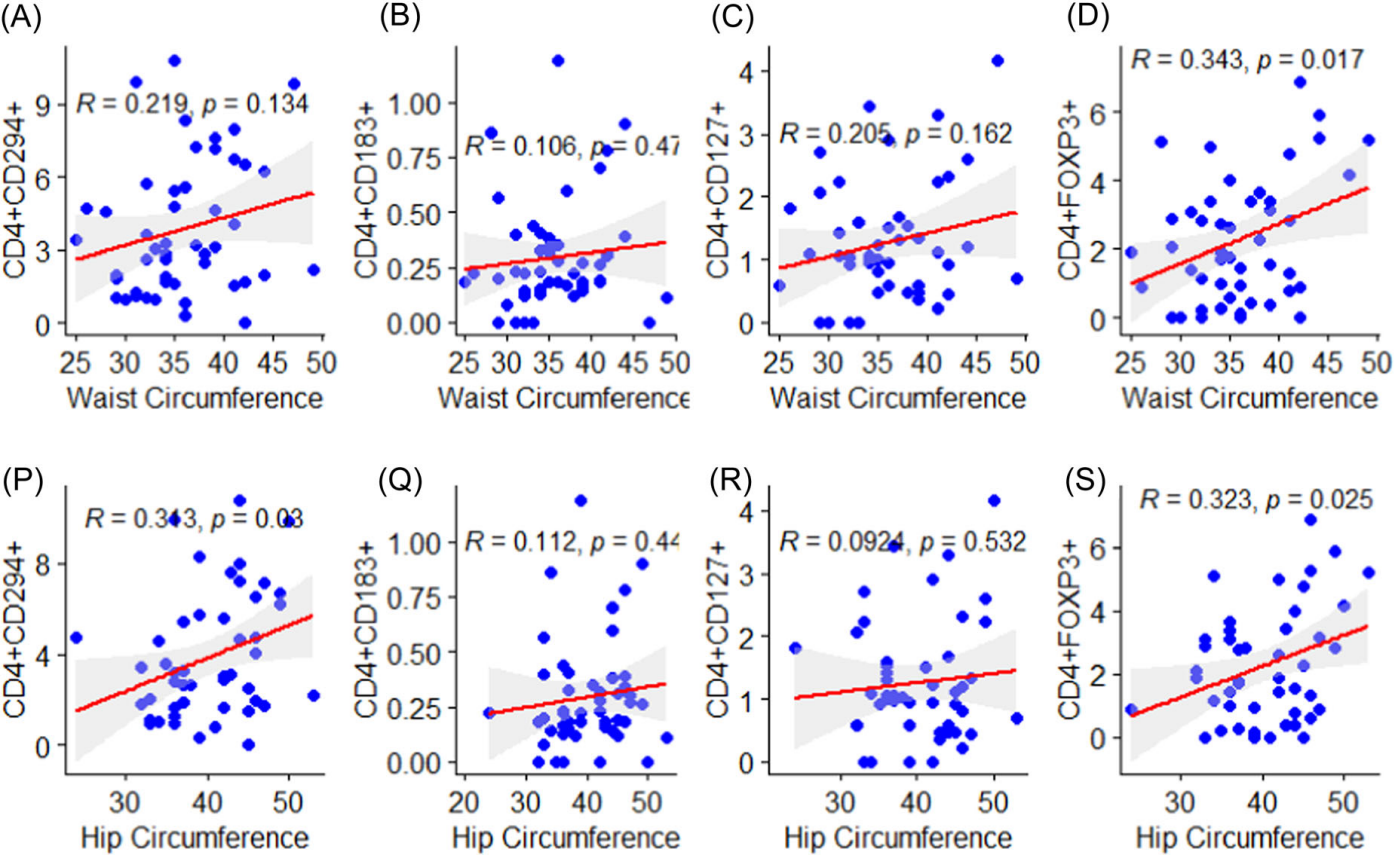
Depicts the correlation between waist circumference (A–D), hip circumference (P–S) and receptor expressions among rheumatoid arthritis (RA) patients.

There was significant positive correlation between waist circumference and CD4+FOXP3+ (*r* = .343, *p* = .017) (Figure 5D). Although there were positive correlations between CD4+CD294+, CD4^+^CD183^+^, CD4+CD127+ and waist circumference, these were insignificant (*p* > .05) (Figure 5A–C).

Moreover, the study found significant positive correlations between CD4+CD294+ (*r* = .313, *p* = .030), CD4+FOXP3+ (*r* = .323, *p* = .025) and hip circumference (Figure 5P,S). However, there were insignificant positive correlation between CD4^+^CD183^+^ (*r* = .112, *p* = .440), CD4+CD127+ (*r* = .0924, *p* = .532) and hip circumference (Figure 5Q,R).

## 4 DISCUSSION

RA is a world‐wide disease affecting adults with a significant impact on daily lives. Previous studies by Sabi et al. and Sehgal et al. reported hypoxia‐inducible factor and endothelin plays a significant role in the progression of RA.^27^, ^28^ Moreover, chemokine receptors and other relevant biomarkers in RA patients could serve as potential diagnostic biomarkers since they contribute to the increase in disease activity of RA. Additionally, studies have demonstrated that targeting these chemokine receptors can reduce joint inflammation and damage, for example, inhibition of CCR2 has also been shown to reduce joint inflammation in animal models of RA and in human clinical trials. The CD4 cells produce chemokines and express chemokine receptors that have been shown to contribute to chronic inflammation in RA and other autoimmune diseases.^29^ However, the levels of these receptors expression biomarkers in RA patients have produced conflicting results. This study investigated the expression of CD4+CD294+, CD4+CD127+, CD4+FOXP3+, and CD4^+^CD183^+^ in RA patients, and their relationship with disease activity in RA.

In this study, CD4^+^CD183^+^ or CXCR3 levels were significantly lower in RA patients compared with the healthy controls as displayed in Figure 2. Contrary to other studies, CD4^+^CD183^+^ has been reported to be highly expressed in RA.^30^, ^31^ However, Suzuki et al. reported that other chemokines receptors such as CCR5 expression was decreased in RA patients compared with healthy controls, and explained that differential expression of chemokine receptors plays a critical role for selective recruitment of pro‐inflammatory T cells into the joints of RA^32^; which was similar to the results of this study, thus this study is the first to report the low expression of CD4^+^CD183^+^ among RA patients in the Ghanaian population.

Moreover, the present study finding shows that among RA patients with high diseases activity, there was a decrease in the CD4^+^CD183^+^ (CXCR3) levels according to the DAS scores and there was no significant correlation between CXCR3 and DAS score. However, El‐Barbary et al. carried out a study to compare the peripheral blood CXCR3 expression among RA patient with low, moderate and high DAS‐28 scores and to investigate the relationship between disease activity and CXCR3 expression.^33^ They reported that there was an increase in peripheral blood CXCR3 levels in RA patients with high diseases activity and the CXCR3 levels has a positive correlation with DAS‐28 score. Chemokine receptors, particularly CD4^+^CD183^+^ and their corresponding ligands play integral roles in exacerbating chronic inflammation associated with severe RA.^34^ However, in Aeberli et al. study to explore the regulation of factors involved in lymphocyte trafficking in patients with RA undergoing treatment with tumor necrosis factor α (TNF‐α) inhibitors, they reported that there was a robust negative correlation between CXCR3^+^/CD4 T lymphocytes and DAS‐28 after 6‐weeks which appeared to be independent of C‐reactive protein (CRP).^35^ Although this study found a significance difference in the levels of receptor expression of CD4^+^CD183^+^ between RA cases and healthy controls, the decreased levels of CD4^+^CD183^+^ may explain the reason for most RA patients presenting with low to moderate disease activity in this study, and that the weak correlation may be due to the small sample size used in this study. Therefore, a larger sample size is needed in the Ghanaian population compared with other diverse populations that must have found significant relationship between CD4^+^CD183^+^ and disease activity.

Furthermore, this study investigated the diagnostic performance of receptor biomarkers in predicting disease activity among RA patients. From the ROC analysis, only CD4^+^CD183^+^ (CXCR3) could predict the disease activity among RA patients with higher sensitivity; however, had low specificity. In a study by Hamed et al, CXCR3 could diagnose and discriminate RA patients at different points through the course of the disease.^36^ Therefore, the findings from this study supports the diagnostic capacity of CD4^+^CD183^+^ in RA disease. Conversely, the low specificity of CD4^+^CD183^+^ for detecting RA observed in the present study further calls for the need for more studies of other potential biomarkers such as the possible biomedical applications of various nanostructures,^37^ in diagnostics and therapeutic approaches of RA. Previous investigations showed nanomaterials such as niosomes, liposomes, polymeric micelles and polymeric nanoparticles as potential therapeutic approaches in RA.^38^, ^39^, ^40^, ^41^

In addition, the study noted that there was significant positive correlation between waist circumference and CD4+FOXP3+, and a found significant positive correlations between CD4+CD294+, CD4+FOXP3+ and hip circumference. Interestingly, the study also observed a significant difference in the mean waist circumference (cm) and mean hip circumference of RA patients and controls. This study finding is suggestive of variation of these biomarkers in RA due to changes in anthropometric indices. Previous studies have reported being overweight may worsen RA symptoms, increase other health problems and may make certain arthritis medications less effective. Study by de Resende Guimarães et al. found obesity was highly prevalent in RA patients and was associated with disease activity.^42^ Crowson et al. reported obesity is associated with a modest risk for developing RA.^43^ Moreover, obesity was found by Abuhelwa et al. to be negatively associated with RA disease remission regardless of RA therapy, suggesting that baseline of anthropometrics such as BMI should be considered as a stratification factor in future RA trials.^44^ Thus, although, there is no scientific bases for significant correlation between waist circumference and CD4+FOXP3+; and between CD4+CD294+, CD4+FOXP3+ and hip circumference correlation as it the first time being reported in this study, these findings could serve as a primary guide to further studies.

Despite the high CD4^+^CD183^+^ sensitivity as the best receptor biomarker for detecting RA, it was limited by its low specificity. This could be attributed to study's relatively small sample size, nonetheless, it was sufficient to draw immunological conclusions. The low specificity of CD4^+^CD183^+^ for detecting RA further calls for the need to study other potential biomarkers such as the possible biomedical applications of nanomedicine in diagnostics and prognostic approaches in RA. Moreover, a larger sample size is recommended in future studies to make more meaningful justification of CD4^+^CD183^+^ receptor expressions as possible diagnostics biomarker for RA. This study findings however provide foundation for CD4^+^CD183^+^ as a potential reliable diagnostics and prognostics biomarker for RA and disease activity as well as CD4+CD127+, CD4+CD294 and CD4+FOXP3+, since their activities have been demonstrated to contribute to prognostics of RA.

## 5 CONCLUSION

CD4^+^CD183^+^ best predict RA and is positively correlated with disease activity. CD4^+^CD183^+^ could serve as useful diagnostics and disease‐monitoring biomarker for RA; however, it demonstrates low specificity. Future studies should be directed on CD4^+^CD183^+^ and other biomarkers to augment their diagnostics performances and routine management in RA.

## AUTHOR CONTRIBUTIONS

**Samuel Asamoah Sakyi**: Conceptualization; data curation; formal analysis; investigation; methodology; resources; supervision; writing—original draft; writing—review and editing. **Tonnies A. Buckman**: Conceptualization; data curation; formal analysis; investigation; methodology; resources; validation; writing—original draft; writing—review and editing. **Kwame Yeboah‐Mensah**: Conceptualization; investigation; methodology; writing—original draft; writing—review and editing. **Ebenezer Senu**: Conceptualization; data curation; formal analysis; investigation; methodology; resources; validation; visualization; writing—original draft; writing—review and editing. **Alfred Effah**: Conceptualization; data curation; formal analysis; investigation; methodology; resources; software; validation; writing—original draft; writing—review and editing. **Daniel Antwi‐Berko**: Conceptualization; investigation; methodology; writing—original draft; writing—review and editing. **Dzifa Dey**: Conceptualization; investigation; methodology; writing—original draft; writing—review and editing. **Maxwell H. Antwi**: Conceptualization; investigation; methodology; writing—original draft; writing—review and editing. **Joseph Yorke**: Conceptualization; investigation; methodology; writing—original draft; writing—review and editing. **Andy O. Boateng**: Conceptualization; investigation; methodology; writing—original draft; writing—review and editing. **Akwasi M. Addei**: Conceptualization; investigation; methodology; writing—original draft; writing—review and editing. **Muniru M. Tanko**: Conceptualization; investigation; methodology; writing—original draft; writing—review and editing. **Richard Boateng**: Conceptualization; investigation; methodology; writing—original draft; writing—review and editing.

## CONFLICT OF INTEREST STATEMENT

The authors declare no conflict of interest.

## Data Availability

All data generated or analyzed during this study are included in this article and can be requested from the corresponding author.
